# Decoding congenital heart disease: a multi-omic framework for cardiac lineage and regulatory dysfunction

**DOI:** 10.3389/fcell.2025.1659884

**Published:** 2025-09-18

**Authors:** Huasheng Lv, Fengyu Sun, You Chen

**Affiliations:** ^1^ Department of Cardiology, The First Affiliated Hospital of Xinjiang Medical University, Urumqi, China; ^2^ Department of Pediatrics, Xinjiang Medical University, Urumqi, China

**Keywords:** congenital heart disease, cardiac development, single-cell sequencing, spatial transcriptomics, lineage tracing

## Abstract

Congenital heart disease (CHD) is the most prevalent birth defect worldwide, arising from disruptions in the tightly regulated processes of cardiac lineage specification and morphogenesis. Traditional models linking genotype to phenotype have been limited by low resolution and insufficient temporal mapping. Recent advances in single-cell RNA sequencing, spatial transcriptomics, and integrative multi-omics have transformed our understanding of CHD by enabling high-resolution analyses of the cellular origins and regulatory landscapes underlying malformations. This review synthesizes current insights into the developmental trajectories of first and second heart field progenitors, cardiac neural crest cells, and emerging progenitor populations. We highlight how combining genome-wide association studies with single-cell and spatial atlases can map non-coding risk variants to precise spatiotemporal cell states. Additionally, cardiac organoid and engineered developmental models provide innovative platforms for validating gene function and modeling lineage-specific defects in human tissues. Together, these technologies are shifting CHD research toward a mechanistic, cell-type–resolved framework, opening new avenues for precision diagnostics, targeted prevention, and regenerative therapies aimed at restoring normal cardiac development.

## 1 Introduction

Congenital heart disease (CHD) represents the most prevalent class of congenital anomalies, affecting up to 12 per 1,000 live births and over 1.3 million neonates annually (Hasan et al., 2023; Deng et al., 2025). Despite major advances in prenatal screening, paediatric cardiac surgery, and intensive care, CHD still causes more than 200,000 deaths worldwide each year. Notably, nearly two-thirds of these deaths occur during infancy (Torzone and Birely, 2025). The burden is particularly severe in low-resource regions, where delayed diagnosis and limited surgical capacity lead to higher mortality and lifelong morbidity (Zimmerman et al., 2020; Şen and Ay, 2025).

The aetiology of CHD is remarkably heterogeneous. Large chromosomal abnormalities such as trisomy 21, 18 and 13, microdeletions like 22q11.2, and pathogenic variants in key transcription factors (e.g., NKX2-5, GATA4, TBX20) or signalling genes (e.g., NOTCH1, JAG1) account for approximately one-third of cases (Grueso Cerón et al., 2024; Salzillo et al., 2024; Şen and Ay, 2025; Xu et al., 2025). However, whole-exome and genome-wide association studies increasingly indicate that most risk is polygenic, modulated by epistatic interactions, incomplete penetrance, and gene-environment synergy (Gelb, 2022). Maternal conditions such as diabetes and hypoxia, as well as exposure to teratogenic drugs and micronutrient deficiency, have all been implicated as environmental modifiers that exacerbate or unmask genetic susceptibility (Yasuhara and Garg, 2021; Wang et al., 2025). Classic animal models underscore this complexity. For example, identical Nkx2-5 or Tbx1 mutations can produce divergent phenotypes across mouse strains, and even within the same genetic background. This results in a spectrum of septal, conotruncal, and conduction defects (Feng et al., 2022; Spendlove et al., 2022).

Until recently, dissecting such multilayered pathogenic networks relied on bulk tissue analyses that averaged away critical cell-type-specific signals. Single-cell RNA sequencing (scRNA-seq) has fundamentally transformed this landscape by cataloguing the transcriptomic profiles of tens of thousands of individual cells throughout cardiogenesis. This approach has uncovered lineage bifurcations, transient intermediates, and niche-specific gene regulatory circuits that are invisible to bulk assays (Elorbany et al., 2022; Ren et al., 2023). In parallel, advances in spatial transcriptomics have reintroduced anatomical context by anchoring these single-cell identities to precise coordinates within intact tissue sections, thereby revealing morphogen gradients and biomechanical cues that direct patterning and morphogenesis (McKenna et al., 2016; Kalhor et al., 2018). Multi-omic extensions, incorporating chromatin accessibility, DNA methylation, histone modifications, and proteomic layers, now offer a holistic view linking genotype, epigenetic state, and phenotypic output.

These technologies have yielded several transformative insights. First, they have clarified the temporal emergence and spatial segregation of first heart field, second heart field, and outflow tract progenitors in the human embryo. These findings elucidate how differential Wnt, Hippo, Fibroblast Growth Factor (FGF), and retinoic acid signalling converge to sculpt chamber identity and inflow–outflow polarity (Fernandes et al., 2023; Zawada et al., 2023). Second, they have pinpointed cell-type-restricted effects of disease-associated variants. For example, *de novo* NKX2-5 mutations preferentially perturb atrial cardiomyocyte trajectories, whereas TBX1 insufficiency disrupts neural crest contributions to the pharyngeal arch and aortic outflow (Ye et al., 2023; Kathiriya, 2024). Third, integrative analyses of fetal hearts with complex chromosomal rearrangements have revealed widespread but lineage-specific dysregulation of metabolic and cytoskeletal programs that precede overt anatomical defects (Zawada et al., 2023; Kathiriya, 2024). Collectively, these findings shift the conceptual framework of CHD from isolated structural anomalies to dynamic perturbations of lineage specification and tissue crosstalk.

Parallel progress in vitro modelling has provided functional platforms to interrogate these mechanisms. Human-induced pluripotent stem cell-derived cardiac organoids recapitulate early heart field patterning and enable CRISPR-based introduction of patient variants in an isogenic context (Bowling et al., 2020; Cell Editorial Team, 2020; Yao et al., 2022; Wang et al., 2023). Complementary to *in vitro* models, single-cell spatial transcriptomics has been proposed as a crucial technology to dissect the complex cellular ecosystems and pathogenic circuits in cardiovascular development and disease, bridging experimental systems and clinical samples (Han et al., 2023). Engineered heart tissues and bioprinted constructs offer biomechanically tunable environments to study how altered stiffness or flow affects morphogenesis and maturation (Wan et al., 2025). Additionally, computational approaches—including graph-based lineage reconstruction and machine learning-driven variant prioritization—complement wet-lab efforts and accelerate discovery pipelines.

Despite these advances, critical knowledge gaps persist. Key questions include how polygenic backgrounds modulate penetrance of high-impact variants, how maternal-fetal metabolic states influence epigenetic trajectories, and which developmental checkpoints are amenable to therapeutic intervention either *in utero* or post-natally. Bridging these gaps will require integrative strategies that couple high-resolution omics with advanced organoid modelling, *in-vivo* lineage tracing in large-animal systems, and longitudinal clinical cohorts equipped with deep phenotyping and digital-health surveillance.

The present review assembles current evidence on human cardiac lineage architecture, synthesises mechanistic links between its disruption and the spectrum of CHD phenotypes, and evaluates emerging translational avenues spanning AI-guided diagnostics, maternal-fetal precision medicine and regenerative engineering. By reframing CHD as a continuum of lineage mis-specification rather than a static malformation, we aim to provide a conceptual and methodological roadmap that will guide future efforts to reduce the diagnostic gap, refine risk stratification and enable targeted prevention or repair.

## 2 Blueprint of the heart: cardiac progenitor lineages and their molecular logic

**FIGURE 1:**
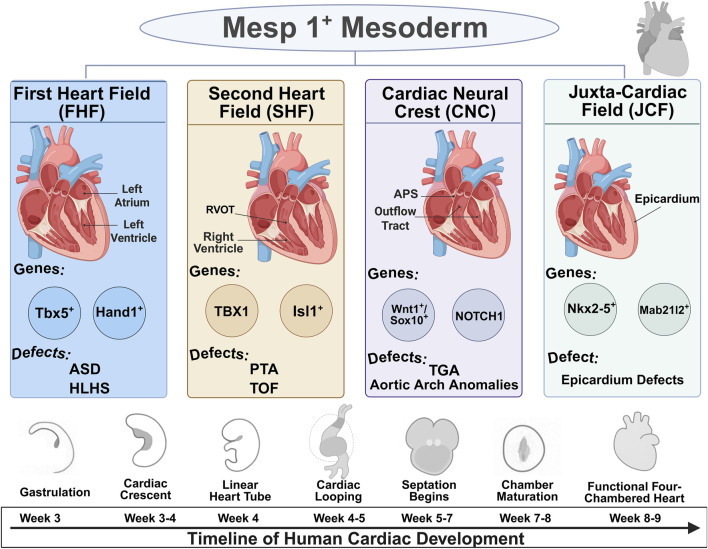
Developmental origins and lineage contributions of cardiac progenitor fields. Schematic illustration of the major cardiac progenitor lineages emerging from Mesp1^+^ mesoderm, including the First Heart Field (FHF), Second Heart Field (SHF), Cardiac Neural Crest (CNC), and Juxta-Cardiac Field (JCF). The timeline below depicts the sequential developmental stages of human cardiogenesis from gastrulation to formation of the four-chambered heart.

The formation of the human heart is a tightly coordinated process governed by discrete progenitor lineages and precisely regulated molecular signals. This section outlines the developmental origins, signaling pathways, and lineage plasticity that constitute the blueprint of cardiac development. Understanding this cellular blueprint, which involves distinct progenitor fields contributing to specific anatomical structures (Figure 1), is fundamental to deciphering the origins of CHD. The key developmental modules, their regulatory networks, and associated disease phenotypes are summarized in Table 1.

**TABLE 1:** Key developmental modules in cardiogenesis and their associated regulatory networks and CHD phenotypes.

Developmental module/lineage	Key regulators (genes and pathways)	Core developmental function	Associated CHD phenotypes/syndromes
FHF	TBX5, HAND1, NKX2-5, GATA4	Specifies LV and atrial identity; drives early septation.	Left-sided defects (e.g., HLHS), Atrial/Ventricular Septal Defects (ASDs/VSDs), Holt-Oram Syndrome.
SHF	ISL1, TBX1, MEF2C, FGF8, Wnt Signaling	Manages progenitor proliferation and multipotency; responsible for forming the RV and OFT.	Conotruncal defects (e.g., TOF, PTA), VSDs, Interrupted Aortic Arch, DiGeorge Syndrome.
CNC and OFT Septation	WNT1, SOX10, PAX3; Relies on SHF-derived signals (Hedgehog, FGF8)	Migrates into the OFT to form the aorticopulmonary septum and the smooth muscle of the great arteries.	Conotruncal defects (e.g., PTA, TGA), Aortic Arch anomalies.
Valvulogenesis (Endocardial Cushions)	Notch Signaling	Governs EMT to form primitive valve cushions and subsequent remodeling into mature leaflets.	BAV, Aortic Valve Stenosis, part of the spectrum in TOF and Alagille Syndrome.
Organ-wide Patterning and Growth	BMP/FGF Signaling, Hippo-YAP Pathway	Regulate cardiac induction, the balance between proliferation vs differentiation, and overall organ size.	Disruption can lead to a wide range of malformations, including hypoplastic chambers (Hippo-YAP loss-of-function).

Abbreviations: FHF, first heart field; SHF, second heart field; CNC, cardiac neural crest; OFT, outflow tract; CHD, congenital heart disease; LV, left ventricle; RV, right ventricle; HLHS, hypoplastic left heart syndrome; ASD, atrial septal defect; VSD, ventricular septal defect; TOF, tetralogy of fallot; PTA, persistent truncus arteriosus; TGA, transposition of the great arteries; BAV, bicuspid aortic valve; EMT, Endothelial-to-Mesenchymal Transition.

### 2.1 Emergence of cardiac progenitors: from mesoderm to heart fields

The heart, the first functional organ to form during embryogenesis, is constructed from a remarkably orchestrated assembly of distinct progenitor cell populations (Paige et al., 2015). These progenitors, originating primarily from the mesoderm, are specified into discrete fields, each with a unique set of molecular markers, developmental potentials, and contributions to the final four-chambered structure. Understanding this cellular blueprint is fundamental to deciphering the origins of CHD.

#### 2.1.1 Early cardiac progenitor specification at gastrulation

Heart development begins soon after gastrulation, when multipotent mesodermal cells are specified into cardiogenic progenitors. A core regulator of this early step is the transcription factor Mesp1, expressed in nascent mesoderm, which acts as a master switch for cardiovascular lineage commitment (Li Y. et al., 2024b). Single-cell lineage tracing and transcriptomic profiling of Mesp1-positive cells have shown that, remarkably, these progenitors are already heterogeneous and pre-patterned toward distinct fates (Li L. et al., 2024). Notably, Notch1 signaling was found to mark the earliest segregation of an endocardial lineage: a subset of gastrulating Mesp1^+^ progenitors expresses Notch1, and fate-mapping showed these cells predominantly populate the endocardium of the mature heart (Lescroart et al., 2018). This defines one of the very first lineage branch points in heart development–the divergence of endocardial *versus* myocardial cell fates–occurring at the mesoderm stage.

#### 2.1.2 The first and second heart fields: distinct origins and contributions

During gastrulation, cardiac progenitor cells migrate to the anterior of the embryo to form a crescent-shaped structure known as the cardiac crescent (Volmert et al., 2023). This structure is not uniform but is composed of at least two major, spatially and temporally distinct progenitor pools: the First and Second Heart Fields (FHF and SHF) (Romero Flores et al., 2023; Chi et al., 2024). Molecular cues help maintain this temporal distinction: for instance, Activin/Nodal and Bone Morphogenetic Protein(BMP) signaling levels have been implicated in biasing mesoderm toward FHF vs SHF fates (Elorbany et al., 2022; Zawada et al., 2023).

FHF comprises the progenitors that differentiate first, folding medially to form the primitive linear heart tube. These cells are fated to become the primary scaffold of the heart, giving rise predominantly to the left ventricle (LV) and contributing to both atria (Galdos and Wu, 2019). The FHF lineage is molecularly defined by the expression of key transcription factors, notably Tbx5 and Hand1 (Zhang Q. et al., 2021; Galdos et al., 2023).

SHF is a population of multipotent progenitors residing in the splanchnic mesoderm medial to the differentiating FHF (Zhao and Yang, 2023). These cells remain in a less differentiated, proliferative state and are progressively added to both the arterial (outflow) and venous (inflow) poles of the elongating heart tube (Volmert et al., 2023). This process of SHF deployment is responsible for the substantial growth and remodeling of the heart, giving rise to the right ventricle (RV), the entirety of the outflow tract (OFT)—which will later septate into the aorta and pulmonary artery—and significant portions of the atria (Galdos and Wu, 2019; Zawada et al., 2023). The quintessential molecular marker for the SHF is the LIM-homeodomain transcription factor Isl1 (Chi et al., 2024).

The sequential and hierarchical nature of FHF and SHF contribution provides a clear developmental logic for the origins of different classes of CHD. The FHF establishes the initial heart tube and LV, while the SHF drives the subsequent expansion and addition of complexity required to form the right ventricle and the great arteries. This temporal distinction means that developmental perturbations occurring at different time points can affect distinct cardiac structures. Defects impacting the FHF are predicted to lead to primary LV anomalies, such as hypoplastic left heart syndrome, whereas disruptions in the SHF progenitor pool are a major cause of right-sided and conotruncal defects (Ward and Kirby, n.d.).

#### 2.1.3 An evolving perspective: the heart fields as a developmental continuum

Beyond the classical view of cardiac lineage segregation, recent studies have begun to redefine this model. While traditionally the heart arises from two major progenitor pools–the FHF and SHF–single-cell analyses suggest these “fields” are initially a continuum of cardiogenic mesoderm rather than rigidly separated lineages. A fate-mapping study showed that at gastrulation/early organogenesis, FHF and SHF cells intermingle as a continuous population of lateral mesoderm, only later diverging into distinct anatomical contributions (Guijarro and Kelly, 2024). This continuum model implies that early cardiac progenitors can be steered into different heart regions by the appropriate signals. Indeed, retinoic acid (RA) signaling has been found to pattern these progenitors along the heart’s anterior-posterior axis. By modulating RA levels in human pluripotent stem cell cultures, researchers can bias differentiating cells toward either FHF-like or SHF-like identities (Zawada et al., 2023; Zhang et al., 2024). This plasticity is further evidenced *in vitro*, where multipotent progenitors can be re-specified by RA or Wnt signals to alter their fate (Snabel et al., 2025).

With the establishment of spatially distinct heart fields, molecular transcription factors come into play to specify lineage fate and regional identity.

### 2.2 Molecular drivers: gene regulatory networks and key transcription factors

The specification and morphogenesis of cardiac lineages are orchestrated by a complex interplay of intrinsic gene regulatory networks (GRNs) and extrinsic signaling pathways. These molecular conductors ensure that progenitor cells proliferate, migrate, and differentiate in the correct place and at the correct time. At the heart of cardiogenesis lies a core network of transcription factors that function as master regulators. This network is highly conserved across species and is characterized by extensive cross-regulation and feedback loops.

A “kernel” of transcription factors, including NKX2-5, GATA4, and TBX5, is essential for initiating and maintaining the cardiac program (Bouveret et al., 2015).

NKX2-5: As one of the earliest markers expressed in cardiac progenitors, Nkx2.5 is considered a master regulator of heart development. It is required for the proliferation of progenitors, the looping of the heart tube, and the establishment of the ventricular gene expression program (Cao et al., 2023). Loss of Nkx2.5 in mice results in an arrested, unlooped heart and early embryonic lethality (Bruneau, 2002). In humans, heterozygous mutations in NKX2-5 are a significant cause of familial CHD, most commonly presenting as atrial septal defects (ASDs) and atrioventricular conduction abnormalities (Behiry et al., 2019; Rozqie et al., 2022).

GATA4 and TBX5: These transcription factors are critical partners of NKX2-5. They physically interact on the promoters of downstream cardiac genes, synergistically activating their expression to drive cardiomyocyte differentiation (Välimäki et al., 2021; Gonzalez-Teran et al., 2022). This combinatorial action is a cornerstone of the cardiac GRN. Reflecting their importance, mutations in.

GATA4 are associated with various septal defects, while mutations in TBX5 cause Holt-Oram syndrome, a condition characterized by upper limb deformities and, most notably, ASDs (El Bouchikhi et al., 2020; Lang et al., 2023).

While the FHF relies heavily on the NKX2-5/GATA4/TBX5 axis, the SHF is governed by a distinct but overlapping GRN. The key regulator of the SHF is ISL1 (Ren et al., 2021; Chi et al., 2024). ISL1 is essential for maintaining the SHF progenitor pool in a proliferative and multipotent state, partly through the activation of downstream targets like Mef2c and Hand2. Beyond proliferation, ISL1 is a key determinant of cell fate within the SHF, promoting a ventricular identity while actively repressing the atrial fate program that is driven by retinoic acid signaling (Quaranta et al., 2018). However, transcriptional regulation alone does not suffice. Cardiogenesis is further sculpted by extrinsic signals from surrounding tissues.

### 2.3 Extrinsic signals: Wnt, BMP, FGF, notch and hedgehog pathways

The intrinsic GRNs are constantly modulated by extrinsic signals from surrounding tissues. These signaling pathways provide the spatial and temporal cues that guide progenitor cell behavior.

Wnt Signaling: The Wnt pathway plays a famously dual and context-dependent role in cardiogenesis. Canonical Wnt/β-catenin signaling is initially active to specify mesodermal cells toward a cardiac fate (Horitani and Shiojima, 2024), but must then be actively inhibited to permit the differentiation of these progenitors into cardiomyocytes (Kreuser et al., 2020). This pathway is subsequently reactivated later in development within the SHF, where it is essential for driving the proliferation of Isl1+ progenitors. In parallel, non-canonical Wnt pathways (e.g., *via* Wnt11) are active and generally promote cardiogenic differentiation and morphogenesis (Akoumianakis et al., 2022; Halmetoja et al., 2022;  Li et al., 2024a; Suzdaltseva et al., 2024). This precise temporal modulation of Wnt signaling acts as a master switch, controlling the timing of progenitor specification, differentiation, and expansion.

BMP and FGF Signaling: Bone Morphogenetic Protein (BMP) and FGF signaling are the primary inducers of cardiogenesis, emanating from the anterior endoderm to pattern the overlying mesoderm (Han et al., 2020; Garcia-Padilla et al., 2022). These two pathways often have opposing but coordinated effects. Generally, FGF signaling maintains cardiac progenitors in a proliferative, undifferentiated state, preventing their premature differentiation (Khosravi et al., 2021). In contrast, BMP signaling typically promotes differentiation into cardiomyocytes (Zhu et al., 2014; Yang et al., 2022). A key example of this antagonism occurs in the SHF, where specific signals like BMP4 from surrounding tissues promote cardiomyocyte differentiation by directly inhibiting the FGF-ERK signaling cascade within the SHF progenitors (Tirosh-Finkel et al., 2010). This dynamic antagonism ensures that a pool of progenitors is maintained while others are progressively deployed to the growing heart.

Notch Signaling: As a key pathway for cell-cell communication, Notch signaling is crucial for mediating cell fate decisions between adjacent cells. Its most well-characterized role in the heart is in valvulogenesis (Wang et al., 2021). Notch signaling is required in endocardial cells of the atrioventricular canal and OFT to initiate an endothelial-to-mesenchymal transition (EMT), a process where endothelial cells transform into migratory mesenchymal cells that populate and form the primitive valve cushions (MacGrogan et al., 2011). Later, Notch continues to regulate the remodeling of these cushions into mature, thin valve leaflets. The clinical importance of this role is profound: mutations in NOTCH1 are the most common single-gene cause of bicuspid aortic valve (BAV), which affects ∼1% of the population and strongly predisposes individuals to calcific aortic valve disease later in life (Irtyuga et al., 2024). Notch is also essential for the process of ventricular trabeculation and myocardial compaction, ensuring the formation of a thick, functional ventricular wall (Luxán et al., 2016).

Beyond these classical pathways, emerging evidence highlights the indispensable roles of the Hippo–Yes-associated protein (YAP) and Hedgehog signaling cascades. The Hippo–YAP pathway functions as a critical organ size regulator by controlling cardiomyocyte proliferation during embryogenesis. In the fetal heart, active YAP in cardiomyocyte nuclei promotes cell cycle activity and myocardial growth, whereas Hippo kinase activation restrains excessive proliferation (Meng et al., 2021; Sakabe et al., 2022). This paradigm is clearly demonstrated in mouse models: deleting Hippo pathway components leads to unchecked cardiomyocyte proliferation and cardiomegaly (enlarged hearts), while conversely, cardiomyocyte-specific loss of YAP causes lethal cardiac hypoplasia with abnormally thin ventricular walls (Rao et al., 2022; Zheng et al., 2022). Therefore, insufficient YAP activity may underlie hypoplastic cardiac chambers, as seen in hypoplastic left heart syndrome, making balanced Hippo–YAP signaling pivotal for achieving proper heart chamber size (Mia and Singh, 2019).

Meanwhile, Hedgehog signaling has emerged as a critical pathway patterning the embryonic outflow tract. Sonic hedgehog (Shh), secreted from the pharyngeal endoderm, provides essential trophic signals to both SHF progenitors and cardiac neural crest cells (CNCCs) (Stefanovic et al., 2021; Seo et al., 2024). This is a classic example of inter-lineage communication, where signals originating from 1 cell type (the endoderm-derived pharyngeal tissue) are required to direct the behavior of other, distinct cell populations (the mesoderm-derived SHF and ectoderm-derived CNCCs). Mechanistically, Hedgehog signals are necessary to maintain the proliferation of SHF progenitors and to support CNCC survival. When Shh signaling is absent or blocked, embryos exhibit a spectrum of severe conotruncal defects mimicking Tetralogy of Fallot and persistent truncus arteriosus (Dyer and Kirby, 2009). Furthermore, Hedgehog activity influences inflow tract and septal development; its disruption is tied to atrial septal defects and atrioventricular cushion defects as well (Liu, 2020; Seo et al., 2024). In summary, disruptions in these two pathways can manifest directly in CHD phenotypes such as hypoplastic chambers (Hippo–YAP loss-of-function) and a range of conotruncal and septation anomalies (Hedgehog insufficiency).

Beyond chemical cues, the shaping of cardiac architecture also depends on biomechanical forces exerted within the embryonic heart.

### 2.4 Biophysical cues: mechanical forces and left–right patterning

Beyond soluble morphogens, the intrinsic gene regulatory networks are also profoundly influenced by physical forces. Hemodynamic forces generated by the beating embryonic heart are crucial for early cardiac morphogenesis, particularly in driving valve formation (Li et al., 2020). As the heart begins to beat, endothelial cells sense the shear stress and fluid flow using key structures like primary cilia and mechanosensitive ion channels. This mechanotransduction cascade begins when endocardial cells sense blood flow-induced shear stress, a process mediated by mechanosensitive structures like the Piezo1 ion channel. Activation of these channels by physical force triggers the upregulation of the key transcription factor KLF2. KLF2 then acts as a central downstream effector, directly activating the Notch pathway (da Silva et al., 2024; Shaikh Qureshi and Hentges, 2024). This final step is essential for prompting the endothelial-to-mesenchymal transition (EMT) that forms the primitive valve cushions. This establishes a clear signaling sequence for valvulogenesis: Blood Flow → Piezo1 Activation → KLF2 Expression → Notch Activation (Duchemin et al., 2019). Consequently, the loss of these mechanosensory inputs leads directly to congenital valve defects. Zebrafish studies show that mutating multiple flow sensors (e.g., Pkd2, Trpv4, Piezo1/2) impairs atrioventricular valve elongation, and experimentally induced abnormal intracardiac flow produces even more severe valvular malformations (Juan et al., 2023). These findings indicate that redundant mechanical signaling pathways converge on the Klf2/Notch axis to govern valvular development, with mechanosensors like Piezo1 acting as central modulators of endothelial gene expression under shear stress.

Mechanical forces are similarly integral to chamber morphogenesis and the establishment of left-right patterning. The embryonic left-right organizer uses motile cilia to generate a directional fluid flow, which breaks symmetry and guides the laterality of the heart and viscera. Disruption of this cilia-driven flow, as seen in primary ciliary dyskinesia, randomizes left–right patterning and often causes heterotaxy syndrome, which is associated with complex congenital heart defects (Gabriel and Lo, 2020). In mice lacking motile nodal cilia, the heart tube fails to loop correctly, leading to malpositioned outflow tracts and septation anomalies.

Notably, endocardial cells bear primary cilia that can directly sense intracardiac fluid forces, transmitting shear stress signals to the nucleus and activating Klf2 and Notch in regions of high flow (Djenoune et al., 2022). Disturbing such mechanosensing, for instance in cilia gene mutants, yields a spectrum of CHD phenotypes, including atrioventricular septal defects, valve dysplasia, and myocardial wall thinning (Gabriel and Lo, 2020; Gabriel et al., 2021). In summary, biomechanical cues—from cilia-driven flow setting the left-right axis to wall shear stress modulating gene programs in the endocardium—are indispensable conductors of normal heart development, and their disruption represents a fundamental pathogenic mechanism underlying congenital heart defects. Evidence also points to ciliary signaling (which establishes left-right asymmetry) as crucial: defects in cilia (e.g., in heterotaxy syndromes) result in misexpression of left-right patterning genes and complex CHD including septal defects and great artery transpositions (Wells et al., 2022; Juan et al., 2023).

### 2.5 The cardiac neural crest: a critical migratory population

The heart is not a purely mesodermal organ; its proper development is critically dependent on contributions from the ectoderm-derived Cardiac Neural Crest (CNC) cells. These are a transient, highly migratory, and multipotent cell population that delaminates from the dorsal neural tube, travels through the pharyngeal arches, and populates the developing cardiac outflow tract (Waldo et al., 1999; Keyte and Hutson, 2012).

The role of the CNC is indispensable for the correct patterning and septation of the OFT. CNC cells differentiate into the smooth muscle of the great arteries and form the bulk of the mesenchyme within the truncal and conal cushions, which fuse to create the aorticopulmonary septum that divides the common OFT into the systemic aorta and the pulmonary artery (Keyte and Hutson, 2012). Consequently, experimental ablation or genetic disruption of CNC development leads to a spectrum of severe conotruncal defects, including persistent truncus arteriosus (a complete failure of OFT septation) and anomalies of the aortic arch arteries (Waldo et al., 1999).

The development of the OFT is therefore a co-dependent process, requiring intricate signaling and physical interaction between the mesoderm-derived SHF and the ectoderm-derived CNCs. The SHF provides the myocardial wall of the OFT, creating the structural scaffold, while the CNCs orchestrate its internal septation and patterning. This concept of an “OFT developmental module” explains why defects in one lineage can phenocopy defects in the other (Feng et al., 2022; Zawada et al., 2023; Kathiriya, 2024). For instance, a primary SHF defect can lead to a failure to provide the correct signaling cues for CNC migration and septation, while a primary CNC defect leaves a properly formed myocardial tube unseptated. This interplay is exemplified by genes like TBX1, an SHF regulator not expressed in CNCs, whose mutation nonetheless causes defects that mimic primary CNC disorders due to the disruption of SHF-to-CNC signaling (Calmont et al., 2009).

### 2.6 The Juxta-cardiac field and epicardial origins

The classical FHF/SHF/CNC model is continually being refined by new discoveries that reveal additional progenitor populations and a surprising degree of plasticity. For instance, single-cell atlases and fate-mapping experiments have uncovered a novel progenitor population termed the JCF, situated at the rostral border of the cardiac crescent (Meier et al., 2023). Initially characterized by the expression of markers like Mab21l2 while lacking the core cardiac marker Nkx2.5, the JCF appears to be a common progenitor pool for both cardiomyocytes and the epicardium—the outer layer of the heartt (Tyser et al., 2021; Meier et al., 2023). This finding challenges the long-held view that the epicardium arises exclusively from a distinct structure and suggests a shared origin for myocardial and epicardial lineages.

### 2.7 Emerging lineage plasticity and redundancy

Emerging lineage tracing data also reveal novel sources for cardiac cell types. One striking example is the contribution of cardiac neural crest cells (cNCCs) to cardiomyocytes. Previously, the neural crest was thought to form only non-muscular tissues in the heart, but recent fate-mapping showed a subset of cNCCs differentiating into *bona fide* cardiomyocytes in multiple species (Yan et al., 2021). These cNCC-derived cardiomyocytes integrate in both developing and regenerating hearts and are required for proper regeneration after injury in zebrafish (George et al., 2020). Similarly, the epicardium harbors progenitors with surprising potency. While mammalian epicardium-to-cardiomyocyte conversion is still debated, studies in salamander heart injury models and cardiac organoids are hinting that with specific cues, epicardial cells can attain broader fates than once appreciated (Eroglu et al., 2022).

Finally, the early heart appears to deploy lineage redundancy and compensation. If one population is impaired, others can sometimes fill the gap. For instance, in zebrafish embryonic hearts where ventricle muscle is experimentally ablated, some atrial cardiomyocytes can reprogram and transform into ventricular muscle, partially regenerating the missing chamber (Zhang et al., 2013). This plasticity exemplifies the developmental capacity for cell fate switching when needed. In summary, the period of heart tube formation involves dynamic transient progenitor populations and inter-lineage plasticity that is greater than previously recognized. This flexibility provides a buffer against developmental perturbations but can also be a source of pathology if misregulated. Understanding these new lineage relationships is enriching our view of cardiogenesis, offering clues as to how the embryonic heart can sometimes “rescue” itself from perturbation, and why its failure to do so may result in congenital defects.

### 2.8 Epigenetic and post-transcriptional regulation in cardiac lineage commitment

Beyond genetic and signaling-based regulation, cardiac lineage commitment is also governed by epigenetic and post-transcriptional mechanisms that fine-tune gene expression with high precision. Epigenetic regulators are increasingly recognized in heart development; e.g., histone modification enzymes and chromatin remodelers (such as CHD7, which is mutated in CHARGE syndrome) can affect cardiac crest and SHF gene expression, leading to outflow defects (Juan et al., 2023). The activity of core transcription factors and signaling pathways is further refined by other regulatory layers, including non-coding RNAs.

Long Non-coding RNAs (lncRNAs): These versatile molecules can regulate gene expression through various mechanisms, including acting as scaffolds for protein complexes or as decoys for other molecules (Scheuermann and Boyer, 2013). A compelling example is the human-specific lncRNA.

HBL1 (Heart Brake LncRNA 1), which functions as a crucial negative regulator of cardiomyocyte differentiation. In the cytoplasm, HBL1 acts as a “sponge” for microRNA-1 (miR-1), sequestering it. In the nucleus, HBL1 acts as a guide, recruiting the repressive Polycomb Repressive Complex 2 (PRC2) to key cardiogenic gene loci to silence their expression (Han and Yang, 2021). This dual-compartment mechanism provides a sophisticated brake on the cardiac program, which must be released for differentiation to proceed.

MicroRNAs (miRNAs): These small non-coding RNAs are powerful post-transcriptional repressors that fine-tune the expression of entire gene networks (Lozano-Velasco et al., 2024). They are implicated in virtually every aspect of heart development and disease. For example, miR-130 has been shown to modulate the balance of BMP and FGF signaling during early cardiomyogenesis, demonstrating the intricate cross-talk between signaling pathways and post-transcriptional regulators (Garcia-Padilla et al., 2022). The interplay between lncRNAs and miRNAs, as seen with the HBL1 sponge mechanism, adds yet another layer of complexity to the cardiac GRN (Matkovich et al., 2014).

## 3 From lineage to lesion: developmental mechanisms underlying CHD

Disruption of cardiac progenitor deployment, molecular signaling, or interlineage communication can derail normal heart morphogenesis. This section outlines how such developmental derailments give rise to congenital heart defects. Congenital heart disease arises when the meticulously orchestrated processes of cardiac development are disrupted. By mapping specific defects back to their cellular and molecular origins, we can understand CHD not just as a collection of anatomical abnormalities, but as diseases of developmental lineage and regulation.

### 3.1 SHF and conotruncal defects: disrupted deployment and septation

The SHF is particularly vulnerable to perturbations, as its progressive addition of myocardial cells underlies the formation of the right ventricle and outflow tract. The second heart field is a developmental hotspot for CHD, given its extensive contributions to the right ventricle, the outflow tract, and cardiac septa (Guijarro and Kelly, 2024). Perturbations in SHF progenitor proliferation, migration, or differentiation are a primary cause of conotruncal defects, which account for a significant fraction of severe CHD.

A cardinal example is the role of the transcription factor TBX1 in 22q11.2 deletion syndrome (also known as DiGeorge syndrome) (Théveniau-Ruissy et al., 2008). Haploinsufficiency of TBX1 is the major driver of the cardiovascular anomalies seen in this syndrome (Calmont et al., 2009). Tbx1 is essential for maintaining the proliferation of SHF progenitors (Xu et al., 2004). In its absence, the SHF is underdeveloped (hypoplastic), leading to a shortened and malformed OFT (Ward and Kirby, n.d.). This primary defect in SHF deployment manifests clinically as severe conotruncal malformations, including persistent truncus arteriosus, tetralogy of Fallot, and interrupted aortic arch. Furthermore, detailed analysis has shown that Tbx1 loss disproportionately affects the development of the subpulmonary myocardium (the base of the future pulmonary artery). This not only leads to OFT misalignment but also causes anomalous coronary artery patterning, revealing an unexpected developmental link between the SHF myocardium and the proper positioning of the coronary ostia (Théveniau-Ruissy et al., 2008). However, SHF development does not occur in isolation. Its interaction with the migrating CNC forms a functional developmental module crucial for septation and arch patterning.

### 3.2 CNC–SHF axis and pharyngeal signaling defects

The formation of a properly septated outflow tract and aortic arch system depends on a tightly regulated dialogue between the SHF mesoderm and the migrating cardiac neural crest cells. Disruptions in this communication are a major cause of CHD. The Tbx1 story again provides a key example. Tbx1 is expressed in the SHF and surrounding pharyngeal tissues but not in the CNCs themselves (Rammah et al., 2022; Yamaguchi et al., 2023). However, loss of Tbx1 severely disrupts CNC-dependent structures. This occurs because Tbx1 regulates the expression of signaling molecules within the pharyngeal environment that are required to guide CNC migration and support their survival. These signals include members of the FGF (e.g., FGF8) and Slit/Robo families (Kodo et al., 2021).

This relationship is reciprocal. Experimental ablation of CNCs in animal models leads to a secondary defect in SHF deployment, characterized by reduced addition of myocardium to the OFT. This secondary effect appears to be mediated by the resulting dysregulation of FGF8 signaling in the pharynx (Alfano et al., 2019; Rammah et al., 2022). This highlights the concept of a “cardiocraniofacial developmental module,” where the SHF and CNCs are so functionally intertwined that a primary defect in one lineage inevitably causes a secondary failure in the other, culminating in a shared spectrum of complex conotruncal and aortic arch defects.

While genetic interactions dominate most CHD paradigms, environmental insults—particularly maternal metabolic disturbances—can converge on the same developmental axes.

### 3.3 Gene–environment interaction: maternal diabetes as a model

While genetic factors are paramount, environmental exposures during critical windows of development can also cause CHD, often by impinging on the same core developmental pathways. Maternal pregestational diabetes is a well-established teratogen, increasing the risk of CHD in offspring by three-to five-fold (Ibrahim et al., 2023). The associated defects frequently involve the cardiac outflow tract and septa, pointing towards a disruption of SHF and CNC development (Maduro et al., 2023).

Hyperglycemia is the primary teratogenic agent, and recent studies have uncovered a precise molecular cascade that links high glucose levels to cardiac defects. This research provides a powerful model for understanding gene-environment interactions. The cascade begins with hyperglycemia inducing oxidative stress and altering the epigenome of developing cardiac cells. Specifically, high glucose has been shown to decrease chromatin accessibility at the promoter of Nos3, the gene encoding endothelial nitric oxide synthase (Basu et al., 2017). This epigenetic silencing leads to reduced production of nitric oxide (NO), a critical signaling molecule. The decrease in NO, in turn, leads to the upregulation of Jarid2, a transcriptional repressor. JARID2 then binds directly to the Notch1 gene locus, recruiting repressive histone marks that silence Notch1 expression (Basu et al., 2017).

This mechanism elegantly demonstrates how an environmental stressor (hyperglycemia) can trigger a specific epigenetic and transcriptional cascade that ultimately downregulates a master regulator of cardiac development (Notch1). This effect is particularly detrimental in individuals who may already have a genetic predisposition, such as a heterozygous mutation in NOTCH1 (haploinsufficiency). In these cases, the environmental stress pushes Notch signaling below a critical functional threshold, resulting in a severe cardiac defect (Garety et al., 2021). This model provides a clear, mechanistic explanation for how genetic and environmental factors can converge to cause CHD.

## 4 Linking genotype to cellular phenotype: a multi-omic dissection of CHD pathogenesis

CHD arises from complex disruptions in cardiac development, yet its underlying mechanisms are often difficult to resolve due to the interplay of temporal, spatial, and cell-type–specific factors. With the advent of single-cell and spatial omics technologies, researchers now possess the tools to dissect these intricate processes with unprecedented resolution. This section explores how these innovations bridge the gap between genetic variation and phenotypic manifestation in CHD, integrating transcriptomic, epigenomic, and spatial information to construct a mechanistic framework of disease pathogenesis.

### 4.1 The modern toolkit: charting development at single-cell resolution

Modern single-cell and spatial technologies have revolutionized developmental biology by addressing three fundamental questions: “What cell types are present?“, “Where are they located?“, and “What regulatory programs define their fate?”

ScRNA-seq has enabled unbiased profiling of transcriptional states in thousands of individual cells, revealing a diverse and dynamic landscape of cardiac cell types throughout embryogenesis (Forte et al., 2021; Samad and Wu, 2021). These data have been used to generate comprehensive cell atlases of the fetal heart in both mouse and human models, facilitating lineage tracing and developmental trajectory reconstruction through pseudotime and RNA velocity algorithms (Snabel et al., 2025). Concurrently, single-cell Assay for Transposase-Accessible Chromatin (scATAC-seq) maps chromatin accessibility at the single-cell level, revealing the temporal activation of enhancers and promoters that govern cell identity and state transitions (Ameen et al., 2022; Tan et al., 2023).

Spatial transcriptomics (ST), through platforms such as Visium and Multiplexed Error Robust Fluorescence *In Situ* Hybridization (MERFISH), reintroduces tissue context by measuring gene expression directly within intact tissue slices, enabling cell-type localization and neighborhood mapping in three dimensions (Choe et al., 2023; Nguyen et al., 2025). When combined, these technologies create a unified, multi-dimensional view of heart development, allowing researchers to infer signaling pathways, transcriptional hierarchies, and spatial cell-cell interactions—elements essential for understanding how genetic alterations disrupt normal morphogenesis in CHD. A detailed comparison of the strengths, limitations, and applications of these transformative technologies is provided in Table 2.

**TABLE 2:** Comparison of single-cell and spatial transcriptomic technologies.

Aspect	Single-cell RNA-seq	Spatial transcriptomics
Analysis Unit	• Dissociated individual cells yields a “gene expression profile” per cell. Cells are clustered by similarity to define cell types/states. Spatial information is lost.	• Intact tissue sections yields expression data mapped to original tissue location. Can detect spatial patterns of gene expression and cell–cell proximity *in situ*.
Key Strengths	• High resolution identification of diverse cell types, even rare populations. • Ability to reconstruct developmental trajectories (pseudotime) to infer lineage relationships. • Can reveal cell-type–specific gene regulatory networks and novel markers.	• Preserves tissue architecture, revealing how cells organize into structures (e.g., layered myocardium, cushions). • Identifies spatial gene expression gradients and niches (e.g., signaling centers in the heart). • Enables mapping of ligand–receptor interactions in the native cellular context (who signals to whom).
Key Insights Gained	• Defined early lineage bifurcations (e.g., Mesp1 subsets for endocardium vs. myocardium). • Discovered transitional cell states (e.g., migrating SHF progenitors, cushion pre-EMT cells). • Linked specific transcription factors to lineage outcomes (e.g., Hand2 to OFT myocardium; Pitx2 to SHF lineage timing). • Uncovered gene expression changes in CHD models at single-cell level (e.g., mutant vs. wild-type differences).	• Mapped anatomical “cell communities” in developing human heart (e.g., distinct subpopulations across ventricular wall). • Revealed regional signaling environments (e.g., endocardial cushions have internal molecular regionalization). • Identified new cell types defined by location (e.g., spatially distinct fibroblast or cardiomyocyte subtypes in specific heart regions). • Enabled retrospective spatial mapping of scRNA-defined cells (placing cell types back into a 3D context).
Limitations	• Loses spatial context (must infer location of cells *post hoc*). • Snapshot in time; combining stages can suggest dynamics but true lineage tracing requires other methods. • Large cells (e.g., mature cardiomyocytes) can be under-represented or fragmented in single-cell preps.	• Lower gene throughput (not all transcripts or genes can be measured, depending on method). • Lower resolution in some methods (e.g., spot-based spatial methods capture multiple cells per spot). • Data analysis is complex (must deconvolve cell mixtures and align with histology).
Emerging Advances	• Single-nucleus RNA-seq (snRNA-seq) to profile adult hearts (overcomes isolation difficulty for adult cardiomyocytes). • Multi-omics at single-cell (combining transcriptome with epigenome or proteome in same cell) to link gene expression with regulatory DNA or protein levels in cardiac cells. • *In vivo* lineage tracing with scRNA-seq (using genetic barcodes to record lineage in the transcriptome) to truly map lineage relationships.	• Higher resolution and multiplexing: MERFISH, seqFISH, and other multiplexed *in situ* methods can approach single-molecule sensitivity for thousands of genes in single cells within tissue. • Spatial multi-omics: combining spatial transcriptomics with spatial proteomics or imaging (e.g., co-detecting transcripts and proteins, or transcriptome and chromatin state *in situ*). • 3D mapping: reconstructing 3D heart tissue by stacking multiple spatially-resolved sections, enabling volumetric mapping of the developing heart.

### 4.2 Pinpointing cellular defects in monogenic CHD models

**FIGURE 2:**
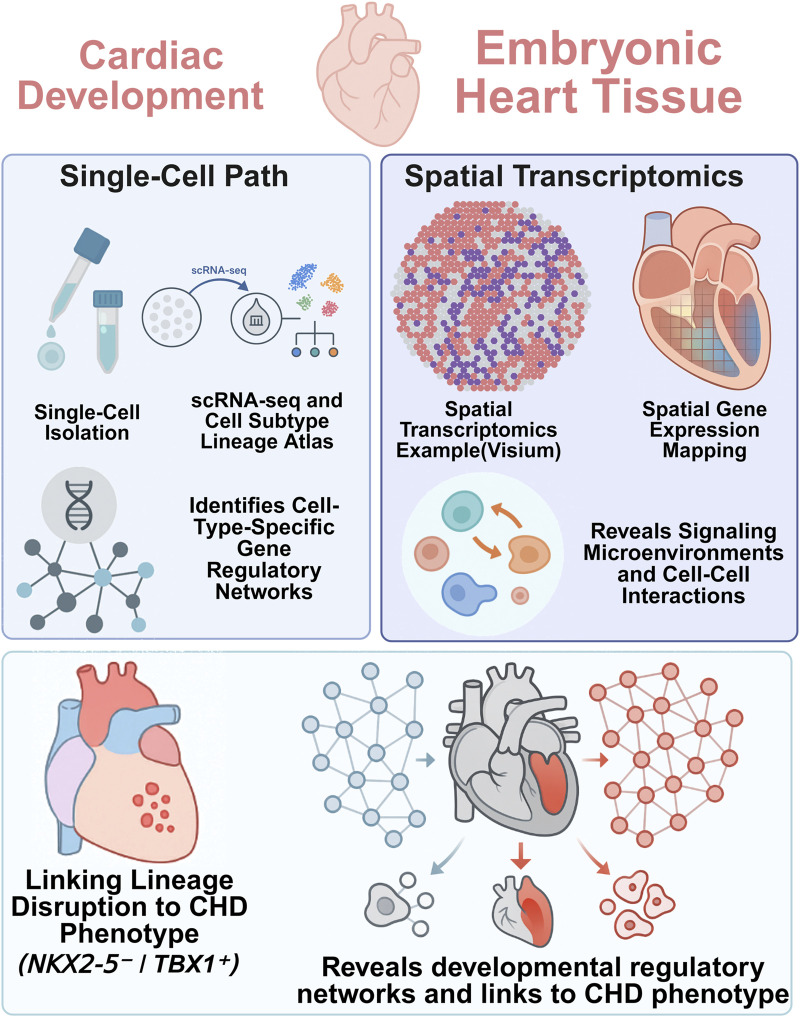
Integrative single-cell and spatial transcriptomics reveal lineage-specific gene regulatory networks.

For monogenic forms of CHD, where specific mutations have been identified, single-cell technologies offer a powerful means to pinpoint the precise cellular and developmental disruptions caused by gene dysfunction. This process, which traces a path from genetic risk to lineage disruption, can be conceptually mapped using an integrated multi-omic workflow (Figure 2).

In Hand2 knockout mouse embryos, for example, De Soysa et al. used scRNA-seq to reveal the selective loss of outflow tract myocardial progenitors, identifying a critical failure in anterior SHF lineage specification (de Soysa et al., 2019). The loss of these progenitors, undetectable by traditional methods, explained the truncation of the OFT phenotype. Furthermore, dysregulated retinoic acid (RA) signaling was uncovered as a major contributing factor, offering mechanistic insight into patterning defects downstream of Hand2 loss.

Similarly, single-cell profiling of Pitx2-null mouse embryos revealed altered differentiation trajectories in both anterior and posterior SHF populations, elucidating how left-right asymmetry defects translate into specific cardiac malformations (Hill et al., 2019). Spatial transcriptomics has also clarified subtle phenotypes, such as compact myocardium thinning in Nfya-deficient models, by revealing spatially restricted proliferation deficits in cardiomyocytes that would be invisible without anatomical context (Li Y. et al., 2024b).

These studies exemplify how single-cell and spatial tools reveal lineage-specific vulnerabilities in monogenic CHD models, tracing the causal chain from gene mutation to disrupted morphogenesis.

### 4.3 Deconvolving polygenic risk: mapping non-coding variants to cell-type-specific enhancers

While monogenic mutations offer tractable entry points, the majority of CHD cases are polygenic, with many risk loci identified by genome-wide association studies (GWAS) lying in non-coding regions of the genome. Deciphering the function of these variants—often labeled as “dark matter”—requires linking them to specific enhancers active in relevant cell types and developmental windows.

scATAC-seq provides the resolution needed to assign enhancer activity to individual cardiac lineages, mapping open chromatin regions across progenitor populations. For example, motifs for key cardiac transcription factors such as NKX2-5, MEF2, and GATA4 have been found enriched in accessible chromatin specific to FHF and SHF derivatives (Ameen et al., 2022). When these regions are intersected with CHD-associated variants, researchers can hypothesize which variants might disrupt enhancer function and in which cell type.

Recent multiomic studies further integrate chromatin accessibility and transcriptional output from the same cell, allowing the construction of cell-type–specific gene regulatory networks (GRNs). In one case, a non-coding variant was found to disrupt a GATA4-bound enhancer active in ventricular cardiomyocytes, and CRISPR-based reporter assays confirmed its loss of function *in vitro*, linking the variant directly to ventricular septal defects (Lage et al., 2012).

**FIGURE 3:**
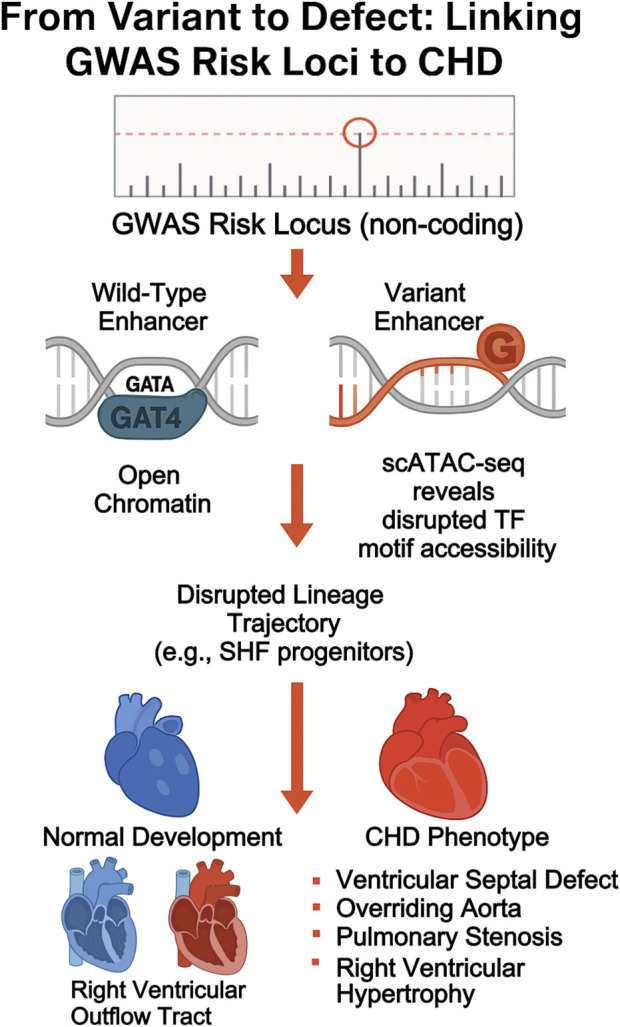
From variant to phenotype: linking non-coding GWAS loci to CHD mechanism. diagram tracing the cascade from GWAS variant to enhancer disruption, transcription factor binding loss, lineage misallocation, and eventual CHD manifestation.

Thus, enhancer-centric maps derived from scATAC-seq and multiome data offer a new lens through which to interpret non-coding GWAS hits, enabling functional annotation and cell-type–specific disease modeling. Figure 3 illustrates this pathway from a GWAS-identified risk locus to the eventual CHD phenotype, while Table 3 provides specific examples of variants linked to cellular and anatomical defects through multi-omic analysis.

**TABLE 3:** Integrative multi-omic findings linking variants to cardiac phenotypes.

Variant/gene	Regulatory element affected	Affected lineage	Resulting CHD phenotype
Enhancer near GATA4	Disrupted TF motif (scATAC-seq)	FHF cardiomyocytes	Atrial Septal Defect
Hand2 coding loss	Loss of OFT trajectory (scRNA-seq)	SHF myocardium	Persistent Truncus Arteriosus
Pitx2-null	Asymmetric SHF deployment	Posterior atrial lineage	Septation defects
TBX1 deletion (22q11.2)	Hypoplastic SHF, impaired CNC signaling	OFT + aortic arches	TOF, Interrupted Aortic Arch

### 4.4 Reconstructing signaling microenvironments in tissue context

CHD is not solely a cell-autonomous phenomenon—it arises in the context of signaling microenvironments shaped by intercellular communication. Spatial omics techniques uniquely enable the reconstruction of these local signaling landscapes.

ST data has revealed morphogen gradients and pathway-specific activation zones essential for cardiac patterning. For example, FGF8 expression is spatially restricted across the OFT, while Notch pathway components are enriched in endocardial cells lining the valves (Asp et al., 2019; Farah et al., 2024). These gradients and compartments are invisible in dissociated single-cell data but become apparent when mapped in spatial context.

Ligand-receptor analysis applied to spatial transcriptomics allows the inference of communication hubs. For instance, atrioventricular endocardial cells were shown to secrete factors that influence adjacent mesenchyme during valve formation, highlighting a critical intercellular axis in morphogenesis (Sylvén et al., 2023; Qiu et al., 2024). Moreover, spatial proteomics techniques such as CO-Detection by indEXing and imaging mass cytometry now allow multiplexed protein-level validation of these interactions *in situ* (Hill et al., 2022; Yao et al., 2023). This reconstruction of the heart’s signaling topology not only explains how local disruptions can cause global defects but also identifies potential paracrine targets for therapeutic intervention in CHD.

### 4.5 Spatially resolved multi-omics: linking genetic risk to spatiotemporal vulnerabilities

The synthesis of scRNA-seq, scATAC-seq, and ST represents a paradigm shift in CHD research, transforming static gene lists into dynamic spatiotemporal risk maps.

A landmark study by Asp et al. produced the first spatially resolved transcriptomic atlas of the human fetal heart, overlaying scRNA-seq data onto anatomical sections to map progenitor niches and developmental trajectories (Asp et al., 2019). Farah et al. expanded this by analyzing over 140,000 fetal cardiac cells using MERFISH, identifying 75 distinct cell types and linking GWAS-prioritized genes to specific regions and lineages—including SHF-derived cells implicated in OFT and septal defects (Farah et al., 2024).

Integrative analyses incorporating chromatin accessibility have shown that many risk variants disrupt enhancers specifically active during key transitions—such as endothelial-to-mesenchymal transformation during valve development. By locating these elements in spatial and lineage context, researchers can now identify the precise “where” and “when” of pathogenesis.

Such insights inform functional experiments. Variants identified in enhancer regions can be validated through induced pluripotent stem cells (iPSCs)-derived cardiomyocyte models, where CRISPR-editing of the candidate locus results in measurable defects in gene expression and cell behavior, directly linking genotype to phenotype.

### 4.6 Limitations and methodological considerations

Despite their transformative power, single-cell and spatial omics technologies face technical and interpretative limitations. Enzymatic dissociation during scRNA-seq introduces stress responses and artificial transcriptional changes, and data sparsity due to gene dropout complicates downstream analysis (Choe et al., 2023). ST platforms, while spatially informative, often lack single-cell resolution, requiring computational deconvolution to infer cell types (Gondal et al., 2024). Lineage tracing methods like CRISPR-based barcoding (e.g., GESTALT, LINNAEUS) offer exciting lineage reconstruction capabilities but suffer from technical challenges such as barcode saturation and homoplasy, which can obscure true clonal relationships (Lopez et al., 2022). Furthermore, multiomic integration demands computational sophistication, and standardized pipelines are still evolving. These limitations underscore the need for careful experimental design, rigorous validation, and orthogonal confirmation using imaging, lineage reporters, or functional assays.

Another significant limitation involves data standardization and reproducibility across studies. Single-cell and spatial transcriptomic datasets often suffer from batch effects arising from differences in tissue processing, library preparation, and sequencing platforms (Lähnemann et al., 2020). These technical variations can obscure biological signals and complicate meta-analyses or cross-cohort comparisons. Moreover, the lack of standardized data formats and sharing protocols hinders the integration of datasets generated by different laboratories. Addressing these challenges will require coordinated efforts to establish robust reference atlases, adopt standardized pipelines for data preprocessing, and implement consensus guidelines for data deposition and sharing.

Finally, translating these technologies into clinical practice raises important ethical and regulatory considerations. The use of fetal tissue samples and *in utero* interventions involves complex ethical frameworks and requires rigorous oversight (Schäffers et al., 2025). Additionally, the clinical utility of *in vitro* disease models and predictive multi-omic signatures must be validated in prospective studies before widespread adoption. Establishing clear regulatory pathways and ethical guidelines will be essential to ensure safe and equitable application of these emerging tools in prenatal diagnostics and targeted therapies.

## 5 Modeling CHD in a dish: from organoids to developmental engineering

The ultimate goal of understanding cardiac development is to translate that knowledge into clinical applications that can prevent or treat heart disease. The emergence of human pluripotent stem cell (hPSC) technology and the field of regenerative medicine have created exciting new avenues to pursue this goal.

### 5.1 Cardiac organoids: a window into human-specific development and disease

Human PSCs, which include both embryonic stem cells and iPSCs, can be coaxed in the laboratory to differentiate and self-organize into three-dimensional structures known as cardiac organoids (Drakhlis et al., 2021; Roshanravan et al., 2023). These “mini-hearts” can recapitulate key features of early embryonic heart development, such as the formation of distinct cell layers and chamber-like cavities, and contain multiple cardiac cell types including cardiomyocytes, endothelial cells, and fibroblasts (Hofbauer et al., 2021; Voges et al., 2023).

**FIGURE 4:**
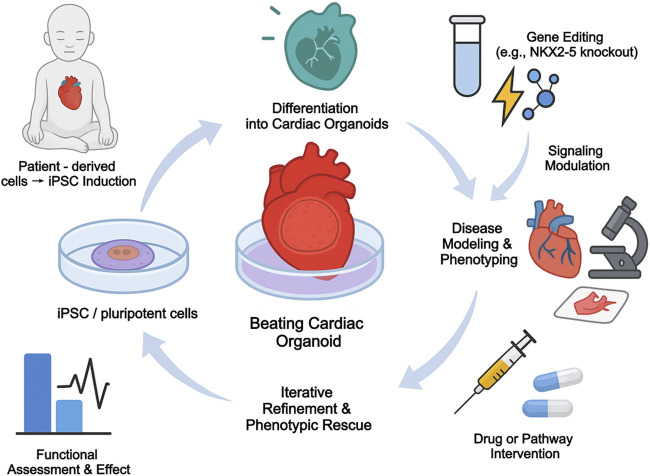
Organoid-based modeling and phenotypic rescue of congenital heart disease.

Cardiac organoids provide an unprecedented *in vitro* platform for modeling human CHD. By creating iPSCs from patients with specific forms of CHD, researchers can generate organoids that carry the individual’s unique genetic makeup. This allows for the study of how a specific mutation disrupts cardiac development in a human context, and provides a platform for testing potential therapeutic compounds (Kathiriya et al., 2021; Mashali et al., 2025). For instance, integrating patient-derived iPSC models of CHD with single-cell profiling has been informative–in one study, iPSCs with a TBX5 mutation (modeling Holt-Oram syndrome) were differentiated to cardiomyocytes and analyzed by scRNA-seq, revealing delayed upregulation of conduction system genes and altered NOTCH signaling in the mutant cells (Kathiriya et al., 2021). Furthermore, organoids can be used to model gene-environment interactions. For example, by exposing developing cardiac organoids to high-glucose conditions, researchers have been able to replicate key cellular and molecular defects associated with maternal diabetes-induced cardiopathy, providing a powerful tool to dissect disease mechanisms (Lewis-Israeli et al., 2021). The general workflow for using patient-derived iPSCs to model disease and test interventions in organoids is shown in Figure 4, and the various applications of different organoid platforms are summarized in Table 4.

**TABLE 4:** Applications of cardiac organoid models in developmental and CHD research.

Model type	Origin	Key features	CHD applications
iPSC-derived cardiac organoids	Human iPSC	Multilineage, chamber formation	TBX5, NKX2-5 loss models
Heart-forming gastruloids	Embryoid body (EB)	Axis patterning, SHF/FHF dynamics	SHF displacement defects
Engineered chamber models	3D printed scaffolds	Contractility, flow simulation	Valve malformation, HLHS
Cardiac-on-chip	Microfluidic culture	Real-time response tracking	Drug screening, pathway inhibition

However, current cardiac organoid models face significant limitations that temper their utility (Yaqinuddin et al., 2025). A major challenge is their structural and functional immaturity; the cardiomyocytes within organoids typically resemble fetal cells rather than adult ones. They often lack a robust, perfusable vascular network, which limits their size and long-term viability (Pang et al., 2021; Landau et al., 2024). They are also missing critical non-myocyte cell populations, such as resident immune cells and autonomic neurons, which are known to play important roles in cardiac function and disease (Thomas et al., 2022). Moreover, many directed differentiation protocols exhibit a strong bias towards generating FHF-derived, left ventricular-like cardiomyocytes, making it difficult to model CHDs that arise from defects in the SHF or other lineages (Williams et al., 2022). Overcoming these hurdles through advanced bioengineering and co-culture techniques is a major focus of the field.

### 5.2 The challenge of cellular immaturity: a unifying hurdle for disease modeling and cardiac repair

The adult human heart has a notoriously limited capacity to regenerate after injury, such as a myocardial infarction, where massive loss of cardiomyocytes leads to the formation of a non-contractile scar and often progresses to heart failure (Fan et al., 2021; Mehanna et al., 2022). Cell-based therapy, involving the transplantation of new cardiomyocytes to replace lost tissue, has long been a major goal of regenerative medicine.

A primary roadblock for this approach has been the profound immaturity of the PSC-derived cardiomyocytes (PSC-CMs) used for transplantation (Guo and Pu, 2020; Kannan and Kwon, 2020). Like the cells in organoids, lab-grown PSC-CMs are metabolically immature (relying on glycolysis rather than fatty acid oxidation), electrophysiologically unstable (exhibiting spontaneous automaticity), and structurally disorganized (Yanamandala et al., 2017). When transplanted into an adult heart, these immature cells integrate poorly with the host myocardium and carry a significant risk of inducing life-threatening arrhythmias (Yanamandala et al., 2017; Guo and Pu, 2020). A deep understanding of the normal developmental cues—including metabolic shifts, mechanical forces, and cell-cell interactions—that drive cardiomyocyte maturation *in vivo* is therefore essential for developing protocols to generate fully mature, safe, and effective PSC-CMs for therapy (Guo et al., 2021; Shewale et al., 2025).

After nearly 2 decades of clinical trials using various cell types (from bone marrow-derived cells to putative cardiac stem cells), the overall clinical benefit of cardiac cell therapy has been disappointingly modest (Banerjee et al., 2018; Broughton et al., 2018; Zhang J. et al., 2021). A key reason is the extremely low rate of cell survival and engraftment; the vast majority of transplanted cells die within days in the harsh, ischemic, and inflammatory environment of the injured heart (Gupta and Losordo, 2010). The small benefits that have been observed are now largely attributed to indirect paracrine effects—where the transplanted cells secrete beneficial factors that support surviving host cells—rather than to true regeneration of new heart muscle (Malliaras and Marbán, 2011).

The challenges facing cardiac organoids and cell therapy are two sides of the same coin: a failure to fully recapitulate the developmental program. The immaturity, lack of cellular diversity, and poor integration seen in these systems all stem from an incomplete understanding of how to orchestrate the complex, multi-lineage process of heart formation. This realization is driving a convergence of the fields of developmental biology and regenerative medicine. The future of cardiac repair is unlikely to involve the simple injection of a single cell type. Rather, success will likely depend on a “developmental engineering” approach. This may involve the co-transplantation of multiple, synergistic cell lineages (e.g., cardiomyocytes with endothelial and epicardial progenitors) to recreate a supportive developmental niche, or the pre-fabrication of vascularized, multi-lineage cardiac patches or organoids for surgical implantation (Mancuso et al., 2020; Sacco et al., 2023). The goal is shifting from simply replacing cells to recapitulating the developmental program that builds functional, integrated, and mature heart tissue.

### 5.3 Current limitations and the path forward: the quest for maturity and complexity

Building upon conventional human pluripotent stem cell (hPSC)-derived cardiac organoids, recent advances have produced next-generation models—particularly epicardioids—that recapitulate more complex tissue architecture and signaling interactions observed *in vivo*. These self-organizing, multilayered constructs contain an outer epicardial layer and an inner myocardial chamber, modeling critical myocardial–epicardial crosstalk during ventricular wall formation. One notable study engineered epicardioids with a laminated, left ventricle–like structure composed of a beating myocardium and overlying epicardium, organized under the control of retinoic acid signaling gradients. These epicardioids mirrored spatial patterning seen in human embryonic development, and single-cell lineage tracing revealed that epicardial cells within these constructs underwent EMT to give rise to fibroblast and smooth muscle–like derivatives, consistent with known *in vivo* epicardial behavior (Sun et al., 2021; Foglio et al., 2024).

These models offer a powerful platform for studying intercellular signaling during cardiac development. Functional perturbation experiments demonstrated that IGF2–IGF1R signaling from the epicardium to myocardium is essential for promoting myocardial growth, and that NRP2 signaling modulates coronary vasculature formation—insights that would be difficult to extract from static embryonic tissue (Meier et al., 2023). The ability to experimentally dissect such dynamic signaling pathways in real time, using human tissue, makes epicardioids a uniquely valuable system for developmental biology.

Cardiac organoids also support patient-specific modeling of congenital heart disease. hPSCs derived from individuals harboring CHD-associated mutations (e.g., in NKX2-5 or TBX1) can be differentiated into cardiac organoids that manifest disease-relevant phenotypes, such as impaired septation, altered electrophysiology, or reduced cardiomyocyte proliferation (Hofbauer et al., 2021). Patient-derived iPSCs carrying congenital heart disease–associated mutations, including TBX1 haploinsufficiency as seen in DiGeorge syndrome, have been used to generate cardiac organoids capable of modeling aspects of disease-relevant phenotypes. Although fully reproducing complex conotruncal defects remains a challenge, these platforms can recapitulate key features of early outflow tract development and myocardial patterning.

Importantly, organoid systems can model polygenic and multicellular cardiac pathologies, not just single-gene disorders. When subjected to hypertrophic stimuli or sarcomeric mutations, epicardioids displayed myocardial thickening, fibrosis marker upregulation, and fetal gene reactivation—hallmarks of hypertrophic cardiomyopathy (Meier et al., 2023). These responses involved coordinated behavior across cardiomyocytes, fibroblasts, and endothelium, supporting the use of organoids as experimental models for complex disease phenotypes. Such models are especially relevant for CHD subtypes involving multiple tissues, such as endocardial cushion defects, which entail interactions between myocardium, endocardium, and neural crest–derived populations.

From a regenerative perspective, these organoid platforms also inform cardiac tissue engineering strategies. Although far from resembling a mature heart, organoids can be used to test cell-based repair approaches—for example, whether epicardial cell sheets enhance myocardial regeneration—or to develop graftable human cardiac patches. Observations of spontaneous self-organization and chamber formation offer clues for refining *in vitro* differentiation protocols, accelerating the generation of functionally mature cardiac subtypes.

In summary, next-generation cardiac organoids, particularly those incorporating epicardial components, provide unprecedented access to human-specific aspects of heart development, congenital malformations, and tissue remodeling. They bridge the gap between molecular understanding and functional modeling, offering platforms for mechanistic investigation, therapeutic testing, and developmentally informed regenerative design—advancing precision strategies for congenital heart disease.

## 6 Future directions and challenges

Congenital heart disease (CHD) arises from disruptions in the finely orchestrated developmental processes that pattern the embryonic heart. This review has traced the path from early cardiac progenitor specification—beginning with the FHF, SHF, and CNC lineages—through the complex interplay of gene regulatory networks, signaling pathways, biomechanical forces, and spatiotemporal cues that sculpt the mature cardiac structure. The clinical manifestations of CHD, from simple septal defects to complex conotruncal malformations, reflect the points at which these developmental pathways go awry.

With the advent of single-cell and spatial transcriptomic technologies, developmental cardiology has entered an unprecedented era of resolution. These approaches have enabled lineage tracing at single-cell resolution, revealed hidden progenitor populations such as the JCF, and uncovered novel modes of lineage plasticity and compensation (Mattoo et al., 2021; Sylvén et al., 2023). At the same time, integration of human genetic studies—from monogenic mutations to polygenic risk scores—has expanded our understanding of CHD from rare gene disruptions to a spectrum of variant burdens that influence risk and phenotype.

Yet, despite these advances, key knowledge gaps remain. The precise molecular mechanisms that regulate multipotency and fate decisions in SHF progenitors remain incompletely understood. The functional role and transcriptional wiring of newly identified cell populations like the JCF require deeper investigation. The challenge of linking patient-specific genetic variants to cellular dysfunction in defined spatiotemporal contexts is far from resolved.

### 6.1 Challenge 1: bridging the scale from cellular defect to anatomical malformation

A primary challenge is to understand how molecular and cellular-level disruptions give rise to macroscopic, three-dimensional anatomical defects. This will require moving beyond static snapshots to dynamic, multi-scale models. A key direction is the construction of spatiotemporal “4D” atlases that integrate spatial transcriptomics, epigenomics, proteomics, and real-time imaging. Such atlases will enable the causal mapping of gene-to-phenotype relationships, revealing how specific genetic perturbations influence developmental dynamics over time. To achieve this, *in vivo* functional validation through CRISPR-based gene editing, optogenetics, and live imaging in vertebrate models will be instrumental. The translucent zebrafish embryo, for example, allows live imaging of heart field migration and heart tube formation, making it an excellent system to observe the consequences of gene knockouts with cellular detail and connect them to morphological outcomes (Farrell et al., 2011; Dogariu et al., 2021; Lowe et al., 2021).

### 6.2 Challenge 2: achieving true fidelity in vitro models

While animal models are crucial, understanding human-specific aspects of CHD requires robust *in vitro* systems. A second grand challenge is therefore the refinement of high-fidelity cardiac models using human pluripotent stem cell (hPSC)-derived organoids. The goal is to create models that more accurately recapitulate chamber patterning, multi-lineage complexity, vascularization, and cell-cell interactions. As obtaining human fetal cardiac tissue for study is difficult, cardiac organoids and human embryoid bodies that mimic aspects of early heart development are clever alternatives (Hofbauer et al., 2021; Voges et al., 2023). When combined with single-cell analysis, they can reveal how specific gene perturbations might lead to human-relevant malformations. This pursuit of higher fidelity will provide superior platforms for both modeling CHD variants and testing the efficacy and safety of therapeutic interventions.

### 6.3 Challenge 3: towards predictive and preemptive medicine

Ultimately, the goal is to predict and prevent CHD. This third challenge involves leveraging computational power to translate our vast developmental datasets into clinical tools. AI-driven machine learning models trained on integrated multi-omic datasets may allow for the early identification of subtle developmental trajectory deviations that are predictive of CHD. This requires a deep understanding of both early and late developmental events. For instance, single-cell and spatial transcriptomics are opening a window into the later stages of heart maturation, such as the formation of the cardiac conduction system or the emergence of metabolic programs. Comparing these normal developmental atlases to pediatric heart disease tissues allows researchers to identify aberrant persistence of embryonic gene programs or a failure to activate maturation programs in disease states (Pachiadaki et al., 2019; Koenig et al., 2022). A single-cell transcriptomic study of failing human ventricular tissue identified cell-type–specific transcriptional programs that closely resembled immature, fetal-like gene expression signatures (Gacita et al., 2021). These findings support the concept that certain pediatric and adult heart diseases may represent a persistence or reactivation of developmental programs, rather than entirely distinct pathological states. By systematically mapping these molecular signatures onto developmental atlases, AI-driven computational models could help detect early deviations from normal maturation trajectories, enabling precision diagnostics and informing individualized therapeutic interventions.

### 6.4 The ultimate goal: a blueprint for diagnosis and repair

The synergy of these approaches will be critical to fill the remaining gaps in our knowledge. This knowledge will not only revolutionize our ability to diagnose and prevent congenital heart disease but will also provide the essential blueprint for engineering functional, mature heart tissue to repair the damaged adult heart. By understanding the normal developmental lineage map, we can refine protocols for regenerative medicine, aiming to generate specific cardiac cell types for therapeutic use (Paik et al., 2020a; 2020b). Ultimately, the integration of developmental atlases with patient data, powered by computational modeling, may enable a form of precision medicine for CHD, where molecular signatures could guide targeted interventions to correct developmental trajectories before a defect becomes irreversible.

## 7 Conclusion

In conclusion, early cardiac development is a story of lineage decisions—cells choosing paths toward becoming parts of a complex organ. By integrating classical embryology with cutting-edge single-cell and spatial genomics, we are writing the most detailed chapter of that story to date. This integrated perspective not only enriches our basic understanding of how the human heart is made, but it is also shedding light on why it sometimes is made with flaws, and how we might fix them. The hope is that continued research at this nexus of developmental biology and genomics will lead to new strategies to predict, prevent, or treat congenital heart diseases, ensuring that more hearts develop with the perfect rhythm of health.
